# Local Suppression of *Dgat2* Augments Fatty Acid Oxidation in Skeletal Muscle in High-Fat Diet-Fed Mice

**DOI:** 10.4014/jmb.2607.07008

**Published:** 2026-09-02

**Authors:** Yeo Jiyun, Ju Young Park, Min Gyeong Kwon, Eun Seong Kim, Rae Hyeon Oh, Byoung Boo Seo, So Young Bu

**Affiliations:** 1 Department of Food and Nutrition, Daegu University, Gyeongsan 38453, Republic of Korea; 2 Department of Animal Resources, Daegu University, Gyeongsan 38453, Republic of Korea; 3 Department of Companion Animal Industry, Daegu University, Gyeongsan 38453, Republic of Korea

**Keywords:** Triglyceride, *Dgat2*, Skeletal muscle, Fatty acids, Glucose

## Abstract

Diacylglycerol acyltransferase 2 (DGAT2) codes an enzyme which synthesize triglyceride by esterifying fatty acid to last portion of diacylglycerol backbone, and contributes to intramyocellular lipid metabolism. Small interfering RNA (siRNA)-mediated knockdown of *Dgat2* was previously shown to reduce AKT phosphorylation and glucose uptake, decrease fatty acid partitioning into triglycerides, and increase free fatty acid release and oxidation in skeletal muscle cells. The current study aimed to determine whether *Dgat2* knockdown affects lipid and glucose metabolism in glycolytic muscle (GM) and oxidative muscle (OM) under high-fat diet conditions, consistent with our previous *in vitro* findings. Male C57BL/6J mice were fed high-fat diet, and treated with *Dgat2* or control-siRNA, and the effects were compared in different muscle types. Muscle *Dgat2* suppression reduced intramuscular triglyceride content by up to 38.2% whereas increased circulating triglyceride levels. In addition, decreased *Gpat3* mRNA levels supported a reduction in lipid esterification capacity. *Dgat2* suppression increased the integration of C14-tagged fatty acids into acid-soluble metabolites, and altered gene expressions related to glucose utilization; GLUT4 protein were decreased and *Pdk2* mRNA increased in both GM and OM fibers. GM exhibited decreased AKT phosphorylation about 50%, whereas OM showed no change in AKT phosphorylation. Noticeably OM exhibited reduced *Hk2* and glycogen accumulation. Together, these findings suggest that muscle *Dgat2* inhibition redirects fatty acid channeling from triglyceride storage toward oxidation *in vivo*, with accompanying changes in glucose metabolism-related markers. These results extend our previous cell-based findings to a more physiologically relevant setting, while highlighting distinct response patterns in GM and OM.

## Introduction

Skeletal muscle significantly influences to overall-body energy metabolism by regulating fatty acid uptake, lipid storage, fatty acid oxidation, and glucose use [1-4]. Skeletal muscle is exposed to an increased flux of fatty acids under conditions of excess lipids such as a high-fat diet. These fatty acids can be stored as intramyocellular triglycerides (TAGs) or disposed of oxidatively [2]. Although accumulation of muscle lipids has often been linked to metabolic dysfunction, neutral lipid synthesis also plays a protective role by clearing excess fatty acids and limiting the formation of potentially-lipotoxic intermediates [2-4]. Thus, factors govern the process of TAG synthesis in skeletal muscles are likely to determine metabolic flexibility and substrate selection.

Diacylglycerol acyltransferases (DGAT) mediate the last stage of TAG synthesis by esterifying diacylglycerol with fatty acyl-CoA. The two major isoforms, DGAT1and DGAT2, appear to compensate for each other but have non-identical metabolic roles in mediating TAG synthesis and subsequent insulin signaling in adipose and liver tissues [5-7]. Reduced *Dgat1* and its enzymatic activity result in a reduction in plasma TAGs, whereas the suppression of hepatic *Dgat1* diminishes liver TAGs in animal models in which dietary fatty acids are delivered to the liver. Tissue-specific inhibition of *Dgat2* in adipocyte and liver also produces a consistent reduction in plasma and liver TAGs. *Dgat2* inhibition causes a decrease in hepatic mRNA levels of lipogenic genes and a reduction in molecular targets of sterol regulatory element-binding proteins, which are involved in *de novo* lipogenesis [8, 9]. Studies in human hepatocytes and skeletal muscle cells have proposed that DGAT2 preferentially channels endogenously-generated fatty acids to process their enzyme activity toward TAGs [6, 10]. DGAT2 inhibition was shown to diminish acetate-derived lipogenesis but increased AKT phosphorylation in human skeletal muscle biopsied in normal, healthy conditions; impaired glucose tolerance; and in athletes [11]. However, the physiological role of *Dgat2* in skeletal muscle under conditions of lipid oversupply is unclear. In particular, it is uncertain whether *Dgat2* plays a functionally essential role in muscle TAG metabolism when dietary fat availability is high.

Our previous study reported that *Dgat2* inhibition in cultured skeletal muscle cell lowered fatty acid partitioning into TAGs, increased intracellular free fatty acids, but diminished AKT phosphorylation and glucose uptake. These effects were observed under high-fatty-acid conditions designed to mimic the lipid-rich environment commonly associated with metabolic disease [12]. However, how *Dgat2* inhibition influence tissue-level fatty acid flux, circulatory lipid, and carbohydrate metabolism is unknown. Because glycolytic muscle (GM) and oxidative muscle (OM) differ in their capacities for lipid handling and glucose metabolism [13-15], the metabolic consequences of *Dgat2* inhibition in a high-fat environment may also differ between GM and OM; however, this possibility has not been investigated. In addition, because *Dgat2* is being studied as a potential therapeutic target for attenuating metabolic disorders, the unanswered concerns regarding *Dgat2* function are especially relevant [16].

Although the inhibition of TAG synthesis may be beneficial in some tissues such as the liver [8, 16], the consequences of *Dgat2* inhibition in skeletal muscles may be more complex. In muscles, TAG synthesis may not only reflect lipid accumulation, but may also function as a buffering pathway that helps to safely sequester excess fatty acids [3, 17]. Therefore, suppression of *Dgat2* could redirect fatty acid metabolism toward oxidation and alter glucose-handling pathways.

The present study investigated whether inhibition of *Dgat2* in mice fed a high-fat diet recapitulated the metabolic alterations which has been previously observed in skeletal muscle cells [12], and whether lipid metabolism altered by *Dgat2* suppression was associated with gene expression related to lipid partitioning and carbohydrate metabolism in GM and OM of animals fed high-fat diet. Muscle *Dgat2* inhibition decreased intramuscular TAG content, increased fatty acid oxidative flux, elevated circulating TAGs, and attenuated the changes in markers related with glucose-handling processes with these changes being observed to different extents in GM and OM. These findings support a model in which *Dgat2* acts beyond TAG synthesis, and regulate fatty acid oxidation and glucose utilization in skeletal muscles under high-fat diet.

## Methods

### Animals and Maintenance

Eight-week-old C57BL/6J male mice were kept under controlled condition which engage 12 h:12 h light–dark cycle and appropriate temperature (20–22°C). A total of 12 mice were initially randomly allocated to either the control (n=6) or *Dgat2*-suppression (n=6) group. The group assignments were subsequently adjusted, as needed, to achieve comparable body weights between the groups. Mice administered either control or *Dgat2* siRNA were fed a high-fat diet (Harlan Teklad, TD.06415, USA); 45% kcal from fat, 36% kcal from carbohydrate, 19% kcal from protein, ad libitum for 14 d and accessed to water without restriction. Then, a transient *Dgat2*-knockdown animal model was developed [17]. At the end day of the experiment, mice were fasted for 3-4 h prior to sacrifice, with free access to water. Mice were anesthetized and sacrificed, and blood and tissue samples were collected immediately. The muscle tissues on the legs were separated into glycolytic muscle (GM) and oxidative muscle (OM) upon dissecting the muscle. For the GM, which contains a lot of type-II-rich fibers [14, 15], the vastus lateralis and white head of the gastrocnemius were collected. For the OM, which has an abundance in type-I fibers [14, 15], the red fiber areas of the gastrocnemius and soleus muscles were dissected; muscle tissues were then divided into portions, and one portion was minced on ice for further analysis for fresh tissues. All collected tissues including remaining skeletal muscles were rapidly excised, snap-frozen in liquid nitrogen and stored at -80°C until further analysis. The animal protocols were approved by the Institutional Animal Care and Use Committee of Daegu University (DUIACC-2022-1-0301-001).

### Atelocollagen-Based siRNA Administration

**Fig. 1:**
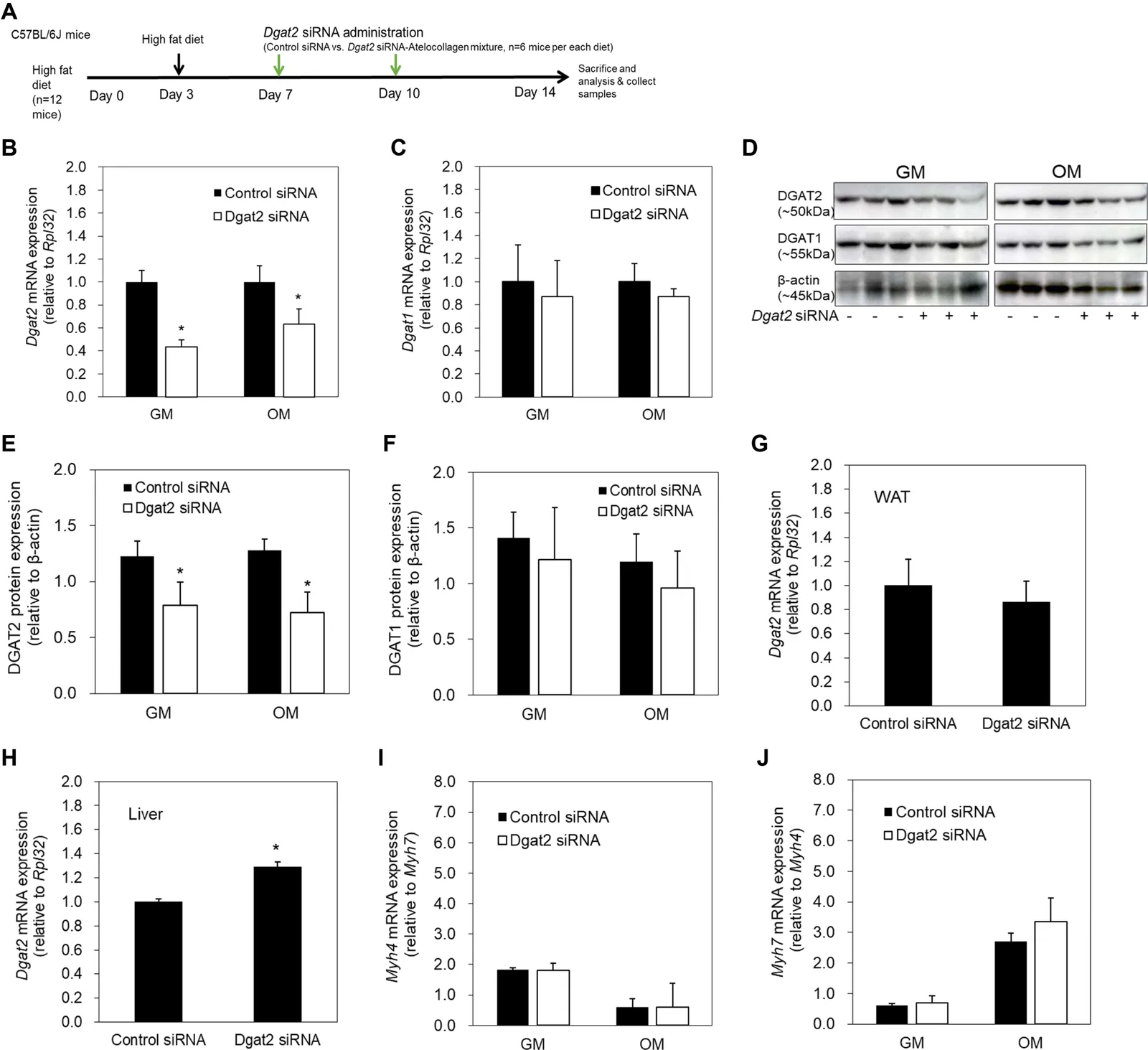
The *Dgat2* expression is reduced by administration of a *Dgat2*–small interfering RNA (siRNA) complex. C57BL/6J mice were fed high fat diet, and received the atelocollagen mix with the control or *Dgat2* siRNA (n = 6 animals/group) as described in (**A**) the schema of the study. (**B**–**F**) Expression level of *Dgat2* and *Dgat1* was measured in mRNA and protein of GM and OM of skeletal muscle (n = 5–6 animals/group). (**G**, **H**) *Dgat2* mRNA was measured in adipose and liver tissues (n = 5–6 animals/group). (**I**, **J**) Expression of *Myh4* and *Myh7* was measured to confirm muscle fiber collection. Although GM and OM are shown in the same panel, statistical analyses were conducted separately for each muscle type. *p < 0.05, vs. control siRNA; GM, glycolytic muscle; OM, oxidative muscle; WAT, white adipose tissue

The *Dgat2* siRNA was designed to target nucleotide positions 498–518 of the registered sequence (NM_026384.3), and the control siRNA was a manufacturer-designed sequence with no homology to any known mammalian gene (Qiagen, USA). Atelocollagen, which have been used to develop gene-suppressing animal models [18, 19], were complexed with siRNAs following the manufacturer’s instruction (Koken Co., Ltd., Japan). A mixture of atelocollagen in QG buffer (AteloGene^®^ Local Use, Koken Co., Ltd, Japan), and siRNA solution (10 μM) was prepared at a 9:1 volume ratio, and mixed by rotation at 4°C for 20 min. The complex was then kept at less than 4°C prior to intramuscular injection. The final injection volume was 180–200 μL per leg, and the mixture was slowly administered by multipoint injection at 3-4 sites in the hindlimb for each treatment group. Mice were administered the atelocollagen-siRNA complex on days 7 and 10 of the experiment. A schematic illustration of the animal investigation is presented in Fig. 1A. Three days after the second siRNA injection (day 14), serum and tissues were collected.

### Real-Time Polymerase Chain Reaction (RT-PCR) and Quantitation

Total RNA was extracted from skeletal muscle fibers according to a commercial protocol (TRIzol reagent, ThermoFisher Scientific, USA). After the quality of RNA was verified using the ratio of absorbance values at 260 nm and 280 nm, a 1 μg of RNA was reacted with random hexamer primers and reverse transcriptase to produce copy DNA (cDNA). Synthesized cDNA was treated with RNAse H, and mixed with SYBR Green-based PCR reagent and probed for the target genes using real-time PCR (Takara PCR Thermal Cycler Dice Gradient, Takara Korea Biomedical, Republic of Korea). The reagents used for cDNA synthesis and real-time PCR were purchased from Takara Biomedical. For quantification of mRNA expression, fluorescence emission data from PCR were obtained for 40 cycles of annealing at 60°C. Expression of mRNA were quantified using the difference in cycle threshold between the target gene and *Rpl32* mRNA, and the quantified fluorescence signals of each gene was standardized to the control group. The list of primers for detecting target genes are presented in Table 1.

### Measurement of TAG and Blood Parameters

Whole bloods were centrifuged (3,000 × *g*, at 4°C), and serum TAG were measured (Stanbio Laboratory, USA). Total lipids were extracted from the skeletal muscle, liver, and adipose tissue of mice [18, 20] and resuspended in sample buffer. A commercial kit was used to quantify total TAG (DoGenBio, Republic of Korea). Serum glucose levels were measured using a colorimetric method using a commercial kit (Asan Pharm., Republic of Korea).

### Measurement of Fatty Acid Oxidation

Radioisotope incorporation into fatty acid oxidation products, acid-soluble metabolite (ASM) and CO_2_ were measured by modifying previously-described methods [21, 22]. To obtain crude mitochondria-rich fraction, both GM and OM fibers were minced with scissors and needle-homogenized in a hypotonic buffer containing 100 mM sucrose, 10 mM Tris-HCl, and 1 mM EDTA (pH 7.4). The procedures were conducted on ice-cold temperature. Homogenates underwent centrifugation (420 × *g*, 4°C), and was reacted with 50 μM [1-^14^C]-oleic acids in the tubes containing 200 μL of 70% perchloric acid and trap filter (Whatman No. 4) added with 1 M NaOH for 1 h. The CO_2_-trapped filter paper underwent scintillation counting to quantify the level of ^14^C-labeled CO_2_. The remaining acid solution underwent centrifugation at 12,000 × *g* for 10 min, and the top-part was used to determine the ASM content in liquid scintillation counter (LS6500IC, USA).

### Glycogen Measurement

The glycogen content in the skeletal muscle fibers was measured using a commercial kit. Briefly, tissue (20–30 mg) were homogenized in 1 mL of 25 mM citric acid (pH 4.2) and 50 mM sodium fluoride on ice. Then homogenates were centrifuged at 14,000 × *g* for 10 min and 10 μL of the supernatant were reacted with commercial enzyme for glycogen breakdown (Sigma, USA) for 30 min. Colored products were measured using the colorimetric method, and the glycogen content was quantified using the equation obtained from the value of the glycogen standard.

### Western Blotting

Well-dounced homogenates of either GM or OM were lysed in a solution of 100 mM sucrose, 10 mM Tris-HCl (pH 7.4), 1 mM EDTA, 150 mM NaCl, 0.1% Triton-X, 1% phosphatase inhibitor, and 1% protease inhibitor cocktail (Roche Life Sciences, USA). Homogenates containing 10–20 μg of protein were combined with protein loading mix which included 2% sodium dodecyl sulphate (SDS), 10% glycerol, 1% bromophenol blue, and 15% β-mercaptoethanol in 50 mM Tris (pH 6.8), and boiled at 100°C for 10 min. Denatured protein samples were subjected to sodium dodecyl sulfate-polyacrylamide gel electrophoresis (SDS-PAGE) and transferred to blotting membranes (polyvinylidene difluoride membranes; Millipore Sigma) in an electrophoresis chamber. Ponceau-S reagent was used to stain and check protein bands on the membrane. After blocking with 1% fish gelatin in Tris-buffered saline (TBS, pH 7.4) containing 0.05% Tween 20, the membranes were probed with antibodies against DGAT1 (1:2000, #PA1-16985, Invitrogen, USA), DGAT2, (1:2000, #SC32399, Santa Cruz Biotechnology, USA), GLUT4 (1:2000, #2213S, Cell Signaling Technology, USA), β-actin (1:5000, #A3854, Sigma), p-AKT, and AKT (1:2000, #9271S, #9272S, Cell Signaling Technology). Membranes were then incubated with a horseradish peroxidase-conjugated secondary antibody, and protein bands were detected using an enhanced chemiluminescence reagent. The acquired protein band images (LAS 2000, GE Healthcare, USA) were assessed using ImageJ software (NIH, USA).

### Statistical Analysis

A total of 12 mice were initially randomly allocated to either the control or *Dgat2*-suppression group. The group assignments were subsequently adjusted, as needed, to achieve comparable body weights between the groups. The animal experiment was conducted once using a single cohort of mice. After tissue collection, each assay was performed in at least two separate runs using samples from the same mice. Repeated measurements from each mouse were averaged to obtain a single value; thus, each mouse contributed only one observation to the statistical analysis. The data are presented as mean ± standard error of the mean (SEM). For some markers showing variation in absolute values across independent experiments, data were normalized within each experiment to the corresponding control group, and the normalized values were then combined for analysis. GM and OM were collected from the same animals. Although the data from GM and OM are presented together in the same graph for comparison, statistical analyses were performed separately within each muscle type, and the two muscle types were not pooled as independent replicates. The reported *n* in the figure legend indicates the number of individual animals used for each analysis. All comparisons of control versus *Dgat2* suppression were conducted by Student's *t*-test. The statistical significance of the difference between groups were determined at *p* < 0.05.

## Results

### *Dgat2* Expression Is Reduced by Administration of *Dgat2* siRNA Complexed with Atelocollagen in Skeletal Muscle

To confirm that *Dgat2* was suppressed in skeletal muscle, *Dgat2* expression was measured initially. Atelocollagen-mediated injection of *Dgat2* siRNA suppressed *Dgat2* mRNA expression by 57% and 37% in GM and OM, respectively, whereas *Dgat1* expression did not change substantially (Fig. 1B and 1C). Although *Dgat2* mRNA reduction in the OM was not as high as that in GM, the degree of suppression in DGAT2 protein levels in OM was comparable to that in GM. The protein expression of DGAT1 was not altered markedly by *Dgat2* suppression (Fig. 1D–1F). To assess potential extra-muscular delivery of siRNA, mRNA expression was measured in white adipose tissue and liver tissue. No significant difference in *Dgat2* mRNA expression was observed in white adipose tissue between the control and *Dgat2*-siRNA-injected groups, whereas hepatic *Dgat2* mRNA expression was approximately 29% higher in the *Dgat2*-siRNA-injected group than in the control group (Fig. 1G and 1H).

Because the GM and OM were analyzed separately based on their distinct fiber-type, the indicative marker genes of each muscle fiber were measured. The level of *Myh4* mRNA was highly expressed in GM, whereas the level of *Myh7* was highly expressed in OM, indicating that the oxidative and glycolytic muscles were collected at the proper level (Figs. 1I and 1J).

### Suppression of *Dgat2* Expression Decreases TAG Content in Skeletal Muscle of Mice Fed High-Fat Diet

**Fig. 2:**
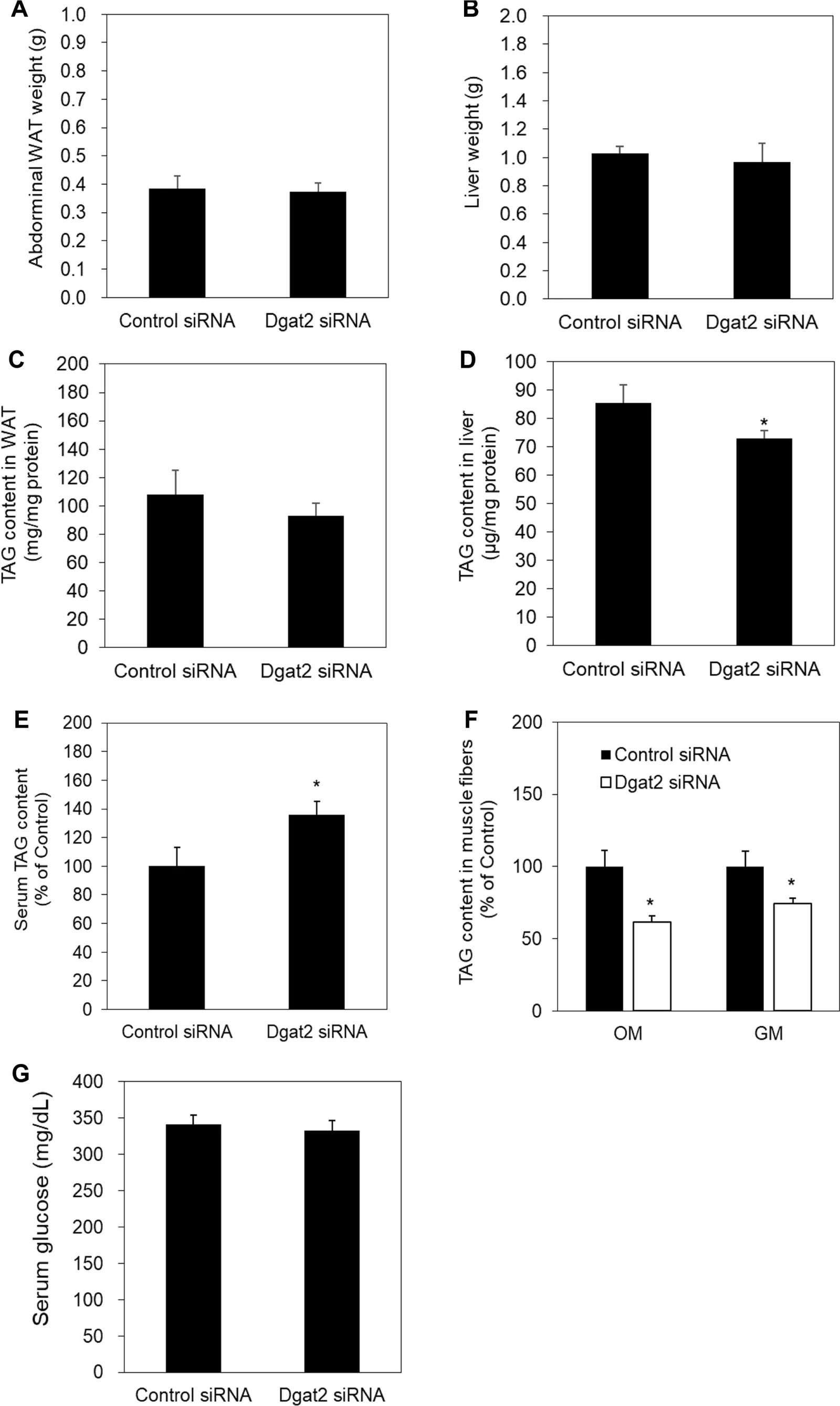
Suppression of *Dgat2* expression decreases TAG content in skeletal muscle. The weight of (**A**) abdominal fat and (**B**) liver were measured. Total lipids were extracted from the collected tissues, and the amount of TAG in the (**C**) adipose tissue and (**D**) liver was measured. The level of (**E**) circulating TAG was quantified. (**F**) The level of TAG was measured in both GM and OM, and (**G**) serum glucose levels were measured. The lipid value was calculated as mg/mg protein and presented as a percentage of the control (n = 4–6 animals/group). Data are presented as mean ± standard error of mean (SEM). Although GM and OM are shown in the same panel, statistical analyses were conducted separately for each muscle type. *p < 0.05, vs. control siRNA; GM, glycolytic muscle; OM, oxidative muscle; TAG, triglyceride; siRNA, small interfering RNA; WAT, white adipose tissue

Body and tissue weights of adipose and liver tissues did not differ between control and *Dgat2* suppressed animals (Fig. 2A and 2B). *Dgat2* suppression in skeletal muscle did not alter TAG accumulation in adipose tissues (Fig. 2C). *Dgat2* reduction significantly decreased TAG in liver (Fig. 2D), but increased serum TAG levels in mice fed a high fat diet (Fig. 2E), which indicate the systemic TAG turnover altered. A decrease in TAG accumulation was observed in both muscle types in response to *Dgat2* suppression. *Dgat2* siRNA decreased the level of TAG accumulation by up to 25.5% in GM and 38.2% in OM of mice fed a high-fat diet (Fig. 2F). *Dgat2* suppression in skeletal muscle did not alter serum glucose levels (Fig. 2G).

### *Dgat2* Reduction Increases Fatty Acid Oxidation in Both Glycolytic and Oxidative Muscle

**Fig. 3:**
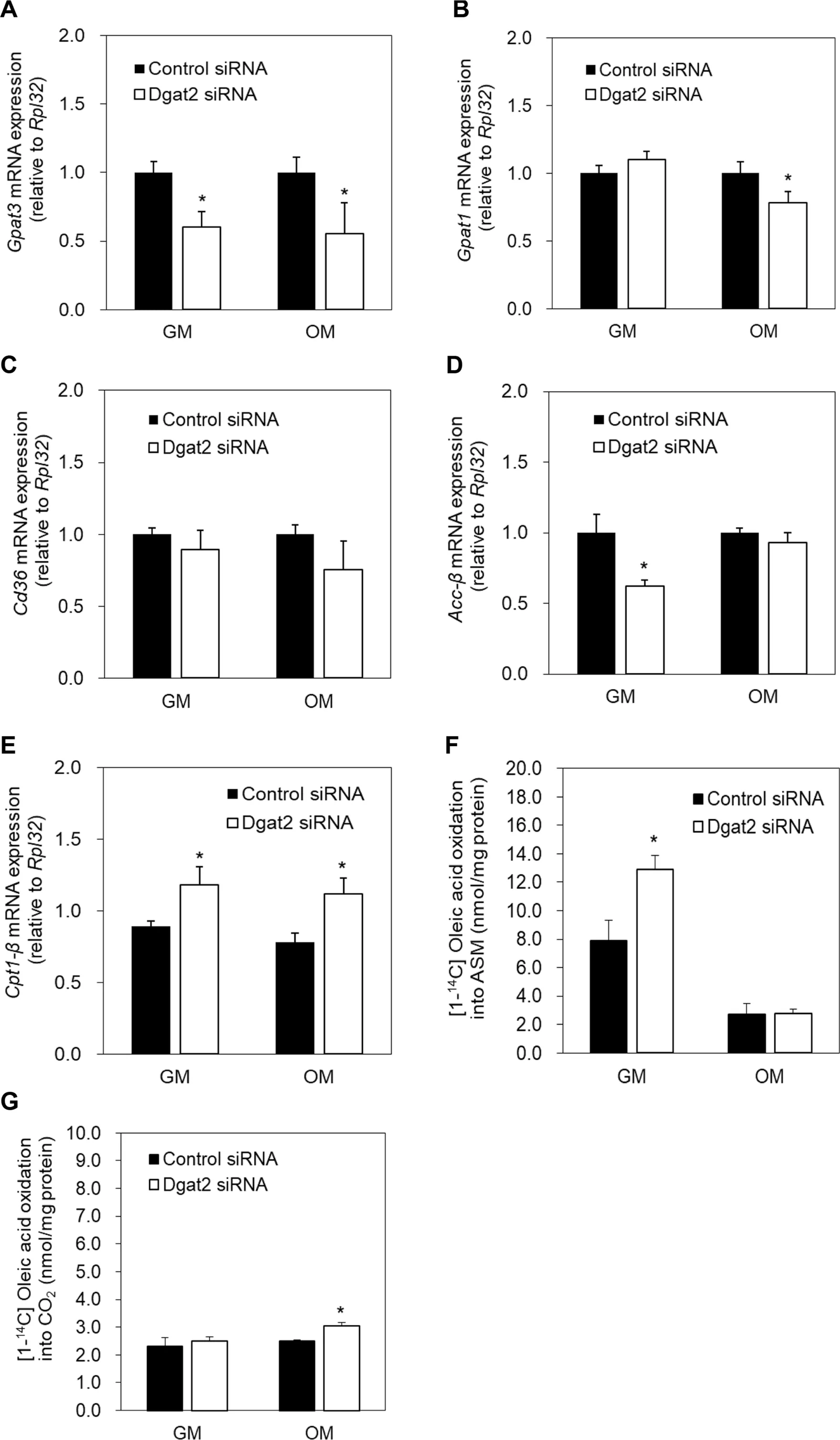
*Dgat2* reduction increases fatty acid oxidation in both glycolytic and oxidative muscle. After trimming skeletal muscle, total RNA was extracted and probed for (**A**) *Gpat3*, (**B**) *Gpat1*, (**C**) *Cd36*, (**D**) *Acc-β*, and (**E**) *Cpt1-β* (n = 4–6 animals/group). Tissues were homogenized in sucrose–Tris–EDTA (STE) buffer and cold oleic acid (50 μM), and [1-^14^C]-oleic acids (0.5 μCi) were added to track fatty acid oxidation. The level of [1-^14^C]-oleic acid incorporation into the (**F**) acid soluble metabolites (ASM) and (**G**) CO_2_ was measured and expressed as nmol of oleic acid per h/mg protein. Although GM and OM are shown in the same panel, statistical analyses were conducted separately for each muscle type. *p < 0.05, vs. control siRNA; GM, glycolytic muscle; OM, oxidative muscle

Based on the decreased TAG levels in skeletal muscle by *Dgat2* suppression, the expression of genes related to lipid metabolism was investigated. Mice injected with *Dgat2* siRNA exhibited decreased *Gpat3* mRNA expression in both GM and OM (Fig. 3A), and decreased glycerol-3-phosphate acyltransferase 1 (*Gpat1*) mRNA only in OM (Fig. 3B). The mRNA levels of and *Cd36* was unchanged (Fig. 3C). The level of acetyl-CoA carboxylase-beta (*Acc-β*) was decreased only in GM, but carnitine palmitoyltransferase 1-beta (*Cpt1-β*) increased in both GM and OM (Fig. 3D and 3E). Fatty acid oxidation rate in the different fibers was analyzed by tracking the conversion of [1-^14^C]-oleic acid into ASM or CO_2_. Both the control and *Dgat2*-suppressed animals had higher [1-^14^C]-ASM than [1-^14^C]-CO_2_. In addition, the extent of ASM increase was pronounced in GM. *Dgat2* suppression increased the level of ^14^C-labeled oleic acid oxidation into ASM by 63.3% in GM, but the amount of ^14^C-labeled ASM was not changed in OM (Fig. 3F). The ^14^C-CO_2_ level was enhanced by *Dgat2* suppression only in OM, but the level was not changed in GM (Fig. 3G).

### *Dgat2* Reduction Alters Glucose Metabolism-Related Markers, with Distinct Patterns Observed in GM and OM

**Fig. 4:**
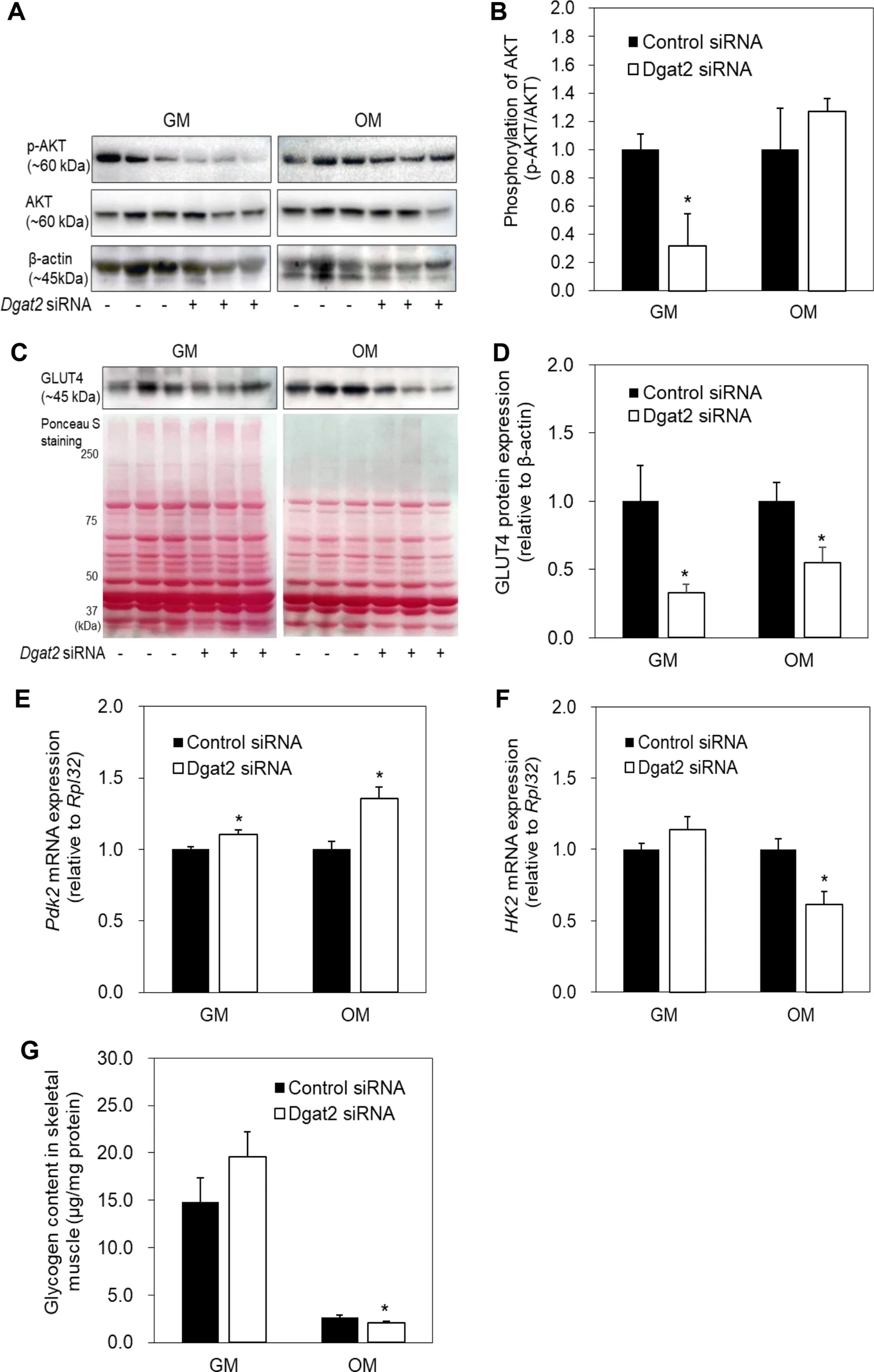
*Dgat2* reduction alters glucose metabolism in different skeletal muscle fibers. (**A-B**) Total proteins were extracted and probed with p-AKT, and AKT, which were measured and quantified on separate membranes; representative images are shown. (**C-D**) The expression of GLUT4 protein was normalized to the total protein amount, as determined by Ponceau S staining (n = 3 animals/group). After trimming the skeletal muscle, total RNA was extracted and probed for (**E**) *Pdk2* and (**F**) *Hk2* (n = 4–6 animals/group). (**G**) Glycogen synthesis was measured in both GM and OM as described in the Methods section (n = 4–6 animals/group). The protein and mRNA expression data were normalized to the expression values for control siRNA. Although GM and OM are shown in the same panel, statistical analyses were conducted separately for each muscle type. *p < 0.05, vs. control siRNA; GM, glycolytic muscle; OM, oxidative muscle

We further measured the expression of carbohydrate metabolism-related genes to investigate whether *Dgat2*-mediated lipid flux was linked to changes in glucose metabolism-related markers. Suppression of *Dgat2* suppressed AKT protein phosphorylation in GM but was not altered in OM (Fig. 4A and 4B). GLUT4 protein expression decreased in both GM and OM (Fig. 4C and 4D). Mice injected with *Dgat2* siRNA exhibited upregulation of pyruvate dehydrogenase kinase 2 (*Pdk2*) in both GM and OM (Fig. 4E). However, the level of hexokinase (*Hk2*) was decreased in OM, but not altered in GM (Fig. 4F). Glycogen content in the OM was decreased by *Dgat2* suppression (29%). The glycogen content between the control and *Dgat2*-suppressed animals was not significantly different in GM (Fig. 4G).

## Discussion

The present study demonstrated that inhibition of *Dgat2* in skeletal muscle decreased the intramuscular TAG content and enhanced fatty acid oxidation. The reduction in intramuscular TAG is consistent with diminished TAG synthesis following *Dgat2* suppression; however, unchanged *Cd36* mRNA expression alone does not exclude a potential change in fatty acid uptake. These changes were coincided with reduced AKT phosphorylation in GM and upregulated *Pdk2* mRNA expression in both GM and OM.

The results indicate that muscle’s energy metabolism shifts to fatty acid oxidation away from carbohydrate utilization by *Dgat2* suppression. Importantly, it should be noted that this study was conducted under high-fat feeding conditions. These data suggest that, under high fat feeding, *Dgat2* functions mainly as TAG-synthesizing enzyme but also regulates indicators related to energy substrate cycling and use in skeletal muscle.

In our previous experiment using C2C12 myotubes, *Dgat2* knockdown impaired insulin-dependent AKT phosphorylation, and glucose uptake, decreased ^14^C-labeled fatty acid partitioning into TAG, but increased intracellular free fatty acids and beta-oxidation [12]. In that study, fatty acids were used at concentrations of up to 500 μM, which represented a relatively high exposure for cultured cells and may be relevant to metabolic disease conditions [12, 23]. A cell culture study, however, did not adequately address these tissue- and circulation-based conditions. The present study extended these findings in physiological condition by demonstrating that similar lipid partitioning pattern under *Dgat2* suppression was preserved in high-fat diet-fed animals. Decrease in intramuscular TAG content and the increase in circulating TAG confirm the concept that skeletal muscle *Dgat2* may function typically as enzyme that esterifies excess fatty acids for storage as proposed by other earlier studies [10, 24], but also regulate the downstream metabolites from diet fat-derived substrates. It should be noted that, in these high-fat-fed animals, the increase in circulating TAG and hepatic *Dgat2* expression, together with the decrease in hepatic TAG following *Dgat2* suppression, suggest that the *Dgat2*-mediated changes observed in this animal model may be related, at least in part, to alterations in hepatic and systemic lipid metabolism. Although skeletal muscle *Cd36* mRNA expression was unchanged, *Cd36* expression alone cannot determine fatty acid uptake or circulating TAG clearance, and these concurrent findings do not establish a mechanistic relationship between skeletal muscle lipid uptake and circulating TAG levels. Therefore, the increase in circulating TAG should be interpreted cautiously. Direct measurements of skeletal muscle fatty acid uptake and circulating TAG clearance are needed to clarify the underlying mechanism.

The increase in fatty acid oxidation following *Dgat2* suppression is particularly noteworthy. Usually, high-fat feeding increases fatty acid delivery to skeletal muscles and induces esterification into TAG or oxidative disposal [2]. The concurrent increase in both the product (*e.g.*, ASM) and gene expression of fatty acid oxidation observed in the current study suggests that when *Dgat2*-mediated TAG synthesis is inhibited, more fatty acids are redirected into oxidative pathways. This is in line with a paradigm from earlier animal studies that showed that *Dgat2* directed fatty acids toward tissue lipid storage in the liver and skeletal muscle tissues [24, 25], and suppression of this pathway forced greater reliance on oxidation, which has been shown in human skeletal muscle [10, 26]. Particularly, in GM which have abundance in fast-twitch muscles [27], *Dgat2* inhibition under a high-fat diet increase gene expression of *Cpt1-β*, which potentially indicate exceeds in mitochondrial oxidative capacity, led to increase of ASMs. The increase in ASM alone suggests that the increase in oxidative flux by *Dgat2* suppression may not necessarily reflect fully-efficient oxidation [28]. This accumulation of incomplete oxidation products might trigger lipotoxicity, selectively impairing AKT phosphorylation in this study. Conversely, in OM which has lots of slow-twitch muscles may boost mitochondrial components accommodates the lipid influx [13, 27], *Dgat2* suppression increased the level of complete fatty acid oxidation to CO_2_. This activation of complete oxidation drives the preventing the accumulation of toxic ASMs and preserving insulin signaling. However, this high rate of fatty acid oxidation in OM was concurred with downregulated *Hk2* expression, and glycogen depletion. Together, these findings suggest that *Dgat2* suppression induces distinct metabolic adaptations in OM and GM, rather than a uniform shift toward improved metabolic function.

The glucose-related modifications identified in this study support the hypothesis of altered fuel selection when TAG synthesis is inhibited. In this high-fat diet-fed mice study, *Dgat2* inhibition decreased AKT phosphorylation in GM and reduced the expression of GLUT4, while increasing *Pdk2*. These results were especially noteworthy for GM, which predominantly uses glucose as fuel [15]. Collectively, these changes are consistent with a metabolic state in muscle that favors fatty acid utilization over glucose use [1]. Although lower expression of GLUT4 and *Hk2* in GM or OM support reduced glucose uptake and intracellular glucose trapping, reduced AKT signaling indicates poor insulin-responsive glucose transport. Increased *Pdk2* expression is also linked to suppression of the pyruvate dehydrogenase-dependent entry of carbon produced from glucose into the tricarboxylic acid cycle [29, 30]. In a study using knockout mice (*Pdhk2*^-/-^, *Pdhk4*^-/-^), *Pdk2* deficiency was shown to increase the activity of pyruvate dehydrogenase complex and lowered the level of blood glucose only in the fed condition, with no change occurring in the fasted condition. In addition, *Pdk4* deficiency exhibited similar effects in fasted animals, suggesting that *Pdk2* was more involved in the fed state, and *Pdhk4* contribute more to the metabolism in fasted state [31]. Although *Pdk4* is known to play a more active role in skeletal muscle, in mice on a high-fat diet with a normal fasting time (3 h) in this study, *Pdk2* may play an important role. Taken together, these findings suggest that inhibiting *Dgat2* may have redirected intracellular fatty acid handling away from TAG storage and toward greater utilization in skeletal muscle.

Currently it is relatively well-established that TAG synthesis can serve as a protective mechanism that sequesters excess fatty acids and limits cells’ exposure to potentially lipotoxic intermediates [2, 18, 25]. Hence, the decrease in TAGs caused by *Dgat2* suppression in this study may appear favorable at first glance but may in fact be accompanied by metabolic trade-offs, including increased fatty acid oxidation and attenuation of markers associated with glucose-utilization process. Our observations under *Dgat2* suppression are somewhat similar to changes occur in prolonged starvation or exercise conditions [2, 32]. Exercise increases fatty acid transport on sarcolemma membrane and make TAG, and TAGs are promptly converted into fatty acids, becoming available substrate for mitochondrial oxidation [32]. Animal study with perilipin (*Plin*)-5 knockout mice fed high fat diet revealed the decreased TAG synthesis, and developed insulin resistance [33]. The *Dgat2*-mediated findings in this study are consistent with this concept and suggest that reduced lipid storage in the muscles should not be interpreted as metabolically beneficial.

An additional finding of this study is that the metabolic response to *Dgat2* suppression appeared to differ according to muscle fiber type. In OM, *Hk2* was reduced without a clear change in AKT phosphorylation, and glycogen-related changes were limited. In contrast, GM showed a more pronounced reduction in AKT signaling, which is in line with the data of previous studies. A study with Wistar rats demonstrated that 18-week of high-fat diet caused impairment of glucose transport, and IRS1 and AKT subunit160 phosphorylation only in tibial anterior muscle, mainly composed of type-II fiber, whereas no change occurred in soleus muscle [14]. In addition, a comparison of muscle fibers from obese and type 2 diabetic subjects reported that type I fibers exhibited less AKT phosphorylation than type-II muscle fibers [27]. Subjects with metabolic syndrome bearing severe insulin resistance had substantially fewer type-I fibers in the skeletal muscle tissues than non-diabetic control subjects. In sedentary men and women, the insulin receptors, protein expression of IRS1 and GLUT4, were lowered in type-I muscle fibers of subjects with metabolic syndrome compared to those in healthy controls [15]. The insulin response and endoplasmic reticulum stress-related protein expression differed between GM and OM in mice under acute depletion of the acyl-CoA activating enzyme [13]. In transgenic mice overexpressing *Dgat2* revealed worsened insulin resistance through the increase of ceramides and acyl-CoA molecules, which was more severe in GM muscle [25]. Based on these findings, carbohydrate metabolism in response to *Dgat2* inhibition is not uniform across different skeletal muscle fibers, and rather affected by oxidative capacity, mitochondrial content, substrate preference, and insulin responsiveness of muscle fibers.

The findings of this study provide insight for *Dgat2* substrate selectivity. Early studies revealed that *Dgat1* has more preference on exogenous fatty acids in most tissue, whereas *Dgat2* was appeared to preferentially channel endogenously-generated fatty acids into TAG [10]. Although this study did not include a normal-diet control group, high-fat feeding is generally recognized as a metabolic condition associated with increased exogenous lipid availability to peripheral tissues [25, 34]. Under these conditions, *Dgat2* inhibition markedly altered intramuscular TAG storage and fatty acid oxidation in skeletal muscle. These finding under high-fat diet is somewhat similar to the alterations revealed by other TAG metabolizing enzyme genes [18, 35], but the consequence on insulins signaling or glucose utilization seem to be different. Deleting acetyl-CoA carboxylase (*Acc*)-*β*, which synthesizes malonyl-CoA and inhibits CPT1 enzyme, decreased whole-body fat contents, and increased palmitic acid oxidation and UCP protein expression in soleus and liver of high-fat-fed animals, however these animals maintained normal levels of insulin and glucose, whereas the WT mice became hyperglycemic [35]. On the other hand, decreased TAG synthesis in *Dgat2*-suppressed skeletal muscle in this study contradicts to findings in mice lacking *Dgat2* in adipose tissue; adipocytes specific deletion of *Dgat2* did not alter TAG synthesis and glucose metabolism maintained in regular range in high-fat-fed mice [7]. Muscle-specific silencing of *Acsl1*, an isoform of acyl-CoA synthetase, which channel fatty acids into TAG synthesis, in high-fat diet-fed C57BL/6J mice decreased expression of mitochondrial β-oxidation enzymes, and the content of the short-chain acylcarnitines and acyl-CoAs, consequently led enhanced insulin sensitivity [36]. Similarly, in our previous study with *Acsl6*-knockdown skeletal muscle cells, high concentration of fatty acids treatment led to increased fatty acid incorporation into fatty acid oxidation and diminished AKT phosphorylation [21]. These combined results suggests that *Dgat2* functions adoptively during exogenous lipid excess to eliminate free fatty acids rather than preferentially choosing fatty acid substrates. Because this study did not directly trace the origin of the fatty acids, we cannot conclude which substrate pools rely on *Dgat2* for TAG formation and channeling in skeletal muscle.

These results are also relevant to the ongoing development of DGAT2 inhibitors for the clinical treatment of metabolic disease, particularly dyslipidemia and nonalcoholic fatty liver disease [16, 37]. Inhibition of TAG synthesis may produce beneficial effects in some tissues, particularly in the liver, and this likely underlies the therapeutic interest in DGAT2 as a drug target [37]. However, the findings suggest that, in skeletal muscle, the metabolic consequences of DGAT2 inhibition may be more complex than a uniformly-beneficial reduction in lipid storage. Because skeletal muscle is metabolically geared toward energy expenditure, TAG synthesis in this tissue may function as a fuel sink for excess fatty acids. Inhibition of this pathway may therefore produce a trade-off in which reduced TAG storage is accompanied by increased fatty acid oxidation and attenuation of glucose-handling pathways [2, 3, 21]. Accordingly, the data do not necessarily oppose the therapeutic rationale for DGAT2 inhibition but instead highlight that its metabolic consequences are likely to be tissue-specific.

This study had some limitations. This study focused on the consequences of partial *Dgat2* suppression only in skeletal muscle, and therefore could not define how these local metabolic changes interacted with whole-body insulin sensitivity or other metabolic tissues. Although the alterations in AKT phosphorylation and the expression of GLUT4 protein, *Hk2*, and *Pdk2* are potentially associated with glucose uptake and utilization under *Dgat2* suppression, no direct functional evaluation of these processes was performed in the present study. Future studies should determine whether these skeletal muscle-specific metabolic changes under *Dgat2* suppression influence whole-body glucose tolerance, insulin sensitivity, and metabolic crosstalk with other tissues, particularly the liver and adipose tissue.

Taken together, the present study extends our previous C2C12 cell-based findings [12], by demonstrating *in vivo* that *Dgat2* inhibition alters intramuscular lipid handling and metabolism-related responses under a high-fat diet. In addition, the differing magnitudes of the observed responses in GM and OM suggest that the metabolic consequences of *Dgat2* suppression may not be uniform across skeletal muscles. These observations emphasize the need to assess metabolic adaptations across skeletal muscles when evaluating the impact of *Dgat2* suppression or inhibitor use. However, because the present study examined only a limited set of endpoints, further studies incorporating lipidomic profiling, direct assessment of mitochondrial function, and whole-body energy metabolism will be needed to more fully define the mechanisms linking *Dgat2* suppression to alterations in lipid handling and carbohydrate metabolism in different skeletal muscles.

## Figures and Tables

**Table 1:** List of primer sequences for qPCR.

Gene	Forward primer (5’-3’)	Reverse primer (5’-3’)
*Dgat1*	TTC CGC CTC TGG GCA TT	AGA ATC GGC CCA CAA TCC A
*Dgat2*	GCT GGC ATT TGA CTG GAA CA	AGT AGT CTC GGA AGT AGC GC
*Myh4*	GCT TGA AAA CGA GGT GGA AA	CCT CCT CAG CCT GTC TCT TG
*Myh7*	CTCAAGCTGCTCAGCAATCTATTT	GGAGCGCAAGTTTGTCATAAGT
*Cd36*	TGG AGC TGT TAT TGG TGC AG	TGG GTT TTG CAC ATC AAA GA
*Pdk2*	AAG AGA TCA ACC TGC TTC CTG	GCA TCT GTG AAC TGG CTT AGA G
*Gpat3*	GTA CAT GCC TCC CAT GAC TAG	GAT CCG TTG CCC ACG ATC ATC
*Gpat1*	TCT GCT GCC ATC TTT GTC CAC	TTG GTC TCT TTG AAA ACC CCG
*Hk2*	GCC AGC CTC TCC TGA TTT TAG TGT	GGG AAC ACA AAA GAC CTC TTC TGG
*Cpt1-β*	TTC CGA CAA ACC CTG AAG CT	GGACACAGATAGCCCAGATCTTG
*Acc-β*	ATC ACC ACT CCT TCT GAC CCC ATC	TCT CCA CAG CAA TCA CTC CCA C
*Rpl32*	AAC CCA GAG GCA TTG ACA AC	ATT GTG GAC CAG GAA CTT GC
